# Like a clock in the rabbit's visual cortex

**DOI:** 10.7554/eLife.65581

**Published:** 2021-02-04

**Authors:** Frédéric Chavane, Diego Contreras

**Affiliations:** 1 Institut de Neurosciences de la Timone, UMR 7289, CNRS and the Faculté de Médecine, Aix-Marseille UniversitéMarseilleFrance; 2 Faculté de Médecine, Aix-Marseille UniversitéMarseilleFrance; 3 Department of Neuroscience, University of Pennsylvania School of MedicinePhiladelphiaUnited States

**Keywords:** rules of synaptic connectivity, thalamocortical specificity, fast-spike interneuron, feedforward inhibition, rabbit, Other

## Abstract

Three rules govern the connectivity between neurons in the thalamus and inhibitory neurons in the visual cortex of rabbits.

**Related research article** Bereshpolova Y, Hei X, Alonso JM, Swadlow HA. 2020. Three rules govern thalamocortical connectivity of fast-spike inhibitory interneurons in the visual cortex. *eLife* **9**:e60102. doi: 10.7554/eLife.60102

The cerebral cortex receives thousands of inputs from the sensory organs and uses them to create representations of the world and respond appropriately. With the exception of smells, all sensory signals travel through a part of the brain called the thalamus on their way to the cortex. The principal cells of the thalamus then relay this constant stream of information to the cerebral cortex in an orderly, topographical manner.

When a neuron in the thalamus relays a signal to a neuron in the input layer of the cortex, the cortical neuron receiving the input can be excitatory or inhibitory, changing the nature of the signal. Figuring out the rules that govern the connections between the thalamus and the cortex is fundamental to understanding the transformation of sensory inputs that travel through this route. For example, how is it that activity of the whole visual cortex is driven by input from the thalamus when fewer than 1–5% of the synapses into the primary visual cortex originate in the thalamus (Douglas and Martin, 2004; Markov et al., 2011)? This is only possible if the thalamocortical synapses act cooperatively (Alonso et al., 1996; Swadlow and Gusev, 2001; Bruno and Sakmann, 2006).

Now, in eLife, Yulia Bereshpolova, Xiajuan Hei, Jose-Manuel Alonso and Harvey Swadlow – who are based at the University of Connecticut and the State University of New York College of Optometry – report on how synapses form between the thalamus and the visual cortex in rabbits (Bereshpolova et al., 2020). First, they measured the activity of neurons in the lateral geniculate nucleus (LGN) of the thalamus, which relay visual information, and the response of neurons in the input layer of the cortex (Figure 1A). Based on the neurons’ firing patterns, Bereshpolova et al. established whether specific neurons in the input layer of the cortex were excitatory neurons or inhibitory neurons (which they call suspected interneurons or SINs). Next, they calculated the delay between a neuron firing in the LGN and a neuron responding in the cortex: when a synapse forms between a neuron in the LGN and a neuron in the cortex, this delay should be between one and three milliseconds. With these tools in hand, Bereshpolova et al. moved on to interrogate the cortical circuit to identify other factors that can influence the connectivity between the thalamus and the cortex.

**Figure 1.**
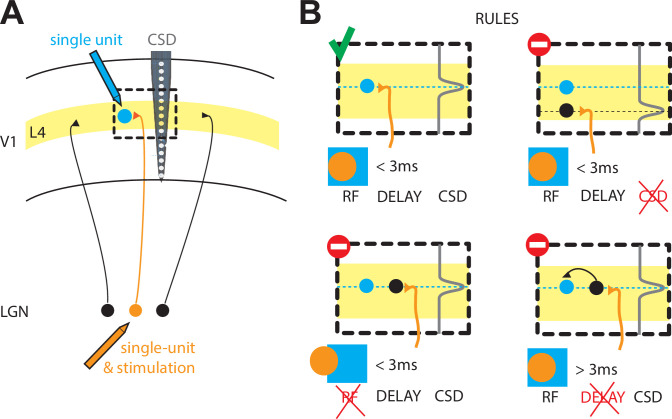
The rules that govern the thalamocortical synapse in rabbits. (**A**) Schematic of the experiments performed by Bereshpolova et al.: a single cell in the LGN of the thalamus (orange) is recorded simultaneously with a single cell (blue) in layer 4 (L4) of the primary visual cortex (V1) during a visual response. The activity across cortical layers is also measured with another electrode (grey, CSD). (**B**) Bereshpolova et al. found that three rules regulate the connectivity between a neuron in the LGN and an inhibitory neuron in L4: their receptive fields (shown here by an orange circle and a blue square respectively) must overlap; the termination of the LGN neuron’s axon (a location estimated by the peak in the CSD signal, grey line) must be located near the cortical neuron (small blue circle); and the delay between the firing of the thalamic and cortical neurons must be less than three milliseconds. All three conditions are met in the top left panel, so a connection is established. Only two of the three conditions are met in the other three panels, so a connection is not established in any of these cases. LGN: lateral geniculate nucleus; CSD: current source density; RF: receptive field.

They found that, similar to cats, the most important condition for a synapse to form was that the 'receptive field' (the region of space in which a stimulus triggers a response) of a neuron in the LGN overlapped with the receptive field of a neuron in the cortex (Figure 1; Tanaka, 1983; Alonso et al., 2001; Sedigh-Sarvestani et al., 2017). Bereshpolova et al. found synapses between 73% of LGN-SIN cell pairs in which more than half of their receptive fields overlapped. LGN neurons could also form connections with excitatory neurons in the cortex, but in this case only 11% of pairs of cells formed synapses when their receptive fields overlapped. Since most of the studied LGN-SIN cell pairs with overlapping receptive fields were connected, this demonstrates that connections between the LGN and SINs are highly promiscuous (SINs receive inputs from almost all the LGN axons in their vicinity).

Why and how are such differences established? The answers are not known, but Bereshpolova et al. were able to explain why the remaining 27% of LGN-SIN pairs with receptive field overlap were not connected. In six of the eleven pairs without connections, the axon belonging to the neuron in the LGN did not terminate close enough to the SIN to form a synapse. This was determined using a laminar electrode (which allows measurements across several cortical layers) and current source density (CSD) analysis (which allows an estimation of the source of a current; Figure 1). In the remaining five pairs, there seemed to be no input from the thalamus into the cortical neurons, since the cortical neurons took over three milliseconds to fire after the LGN neuron had fired. The pattern of cortical neurons receiving input from either the thalamus or from other neurons in the cortex resembles what is seen in the cat visual cortex (Finn et al., 2007).

Despite these similarities between cats and rabbits, there are also differences between the two species. In rabbits, the connections from the thalamus into cortical SINs seem to be much more promiscuous than in cats (Sedigh-Sarvestani et al., 2017). Furthermore, in cats, the vast majority of putative interneurons have receptive fields with regions that respond exclusively to light increase or decrease and do not overlap (so-called simple cells); in rabbits, on the other hand, these two regions do overlap (so-called complex cells). A last important difference is the presence in cats of functional cortical columns, which are absent in rabbits and rodents. These columns are groups of neurons in the cortex that have a similar orientation preference and nearly identical receptive fields, leading to the emergence of orientation maps (regions of the cortex that have similar response properties). These distinctions between cats and rabbits may shed light on how species differ when it comes to thalamocortical connectivity.

The work of Bereshpolova et al. suggests that, in rabbits, a finely tuned clockwork based on three rules governs the connectivity between neurons in the thalamus and inhibitory neurons in the input layer of the cortex: (i) physical proximity; (ii) receptive field overlap; (iii) a short delay (less than three milliseconds) between neurons firing (Figure 1B). The same rules have yet to be explored for the excitatory neurons in the input layer of the cortex (Zhuang et al., 2013). Finally, the differences between the thalamocortical synapses in rabbits and in cats underlines the importance of studying neuroscience in different species.
